# Comprehensive Metabolomic Search for Biomarkers to Differentiate Early Stage Hepatocellular Carcinoma from Cirrhosis

**DOI:** 10.3390/cancers11101497

**Published:** 2019-10-06

**Authors:** Da Jung Kim, Eun Ju Cho, Kyung-Sang Yu, In-Jin Jang, Jung-Hwan Yoon, Taesung Park, Joo-Youn Cho

**Affiliations:** 1 Department of Clinical Pharmacology and Therapeutics, Seoul National University College of Medicine and Hospital, Seoul 03080, Korea; dkim3193@snu.ac.kr (D.J.K.); ksyu@snu.ac.kr (K.-S.Y.); ijjang@snu.ac.kr (I.-J.J.); 2 Department of Internal Medicine and Liver Research Institute, Seoul National University College of Medicine, Seoul 03080, Korea; creatio3@snu.ac.kr (E.J.C.); yoonjh@snu.ac.kr (J.-H.Y.); 3 Department of Statistics, Seoul National University, Seoul 08826, Korea

**Keywords:** hepatocellular carcinoma, cirrhosis, metabolomics, biomarker

## Abstract

The established biomarker for hepatocellular carcinoma (HCC), serum α-fetoprotein (AFP), has suboptimal performance in early disease stages. This study aimed to develop a metabolite panel to differentiate early-stage HCC from cirrhosis. Cross-sectional metabolomic analyses of serum samples were performed for 53 and 47 patients with early HCC and cirrhosis, respectively, and 50 matched healthy controls. Results were validated in 82 and 80 patients with early HCC and cirrhosis, respectively. To retain a broad spectrum of metabolites, technically distinct analyses (global metabolomic profiling using gas chromatography time-of-flight mass spectrometry and targeted analyses using liquid chromatography with tandem mass spectrometry) were employed. Multivariate analyses classified distinct metabolites; logistic regression was employed to construct a prediction model for HCC diagnosis. Five metabolites (methionine, proline, ornithine, pimelylcarnitine, and octanoylcarnitine) were selected in a panel. The panel distinguished HCC from cirrhosis and normal controls, with an area under the receiver operating curve (AUC) of 0.82; this was significantly better than that of AFP (AUC: 0.75). During validation, the panel demonstrated significantly better predictability (AUC: 0.94) than did AFP (AUC: 0.78). Defects in ammonia recycling, the urea cycle, and amino acid metabolism, demonstrated on enrichment pathway analysis, may reliably distinguish HCC from cirrhosis. Compared with AFP alone, the metabolite panel substantially improved early-stage HCC detection.

## 1. Introduction

Hepatocellular carcinoma (HCC) is the fifth most common cancer worldwide and is the major cause of death in patients with chronic liver disease [1,2]. To ensure an early diagnosis and reduction of disease-related mortality, HCC surveillance is essential for at-risk populations [3]. However, tumor biomarkers for the early detection of HCC are scarce. Even α-fetoprotein (AFP), the most commonly used tumor marker, has suboptimal performance, providing only a 6–8% higher rate of detection for HCCs not identified on ultrasound [4]. Therefore, the development of new biomarkers for early diagnosis of HCC is particularly necessary for improving the efficacy of surveillance. 

Global metabolomics—the profiling of metabolites in biofluids, cells, and tissues—have gained popularity for the diagnosis and monitoring of diseases. The identification of numerous metabolites has improved the current understanding of metabolites and systems-level effects, thereby providing novel insights into the underlying mechanisms for various physiological conditions and aberrant processes, including diseases [5]. Metabolomic analysis is extensively employed to develop a set of diagnostic and prognostic biomarkers for various diseases, such as liver disease [5,6,7] and Alzheimer’s disease [8,9]; it is also applied for personalized pharmacotherapy [10]. Since HCC alters various metabolic functions of the liver, metabolomic-based biomarkers may serve as valuable tools for the early detection of HCC. In recent years, a number of metabolomic-based approaches have been applied for HCC. However, most markers were evaluated based on single metabolomic methods, including 1H nuclear magnetic resonance or mass spectrometry-based targeted metabolomics [6,7,11]. As a combination of multiple analytical methods may enable the capture of a broad spectrum of metabolites, we identified HCC-specific metabolite alterations using a combination of gas chromatography (GC)- and liquid chromatography (LC)-mass spectrometry (MS)-based analyses and determined their potential as diagnostic biomarkers for HCC. Quantitation of the significant metabolites established the reliability of the model. This study aimed to develop a biomarker panel of serum metabolites to distinguish early-stage HCC from cirrhosis and provide a pathophysiological interpretation of biomarkers relevant to a disease status. 

## 2. Results

### 2.1. Baseline Characteristics

The baseline characteristics of patients in the training and test sets are summarized in Table 1. Patients in both sets had similar baseline characteristics, except that patients in the validation set were more likely to have hepatitis C virus-related or non-viral liver disease. There were no statistically significant changes in clinical parameters, such as age, sex, and liver function, related to each group. As expected, serum AFP levels were significantly higher in the HCC group than in the cirrhosis and control groups. All patients had very early or early-stage HCC.

### 2.2. Global and Targeted Metabolic Profiling According to Liver Disease

**Figure 1:**
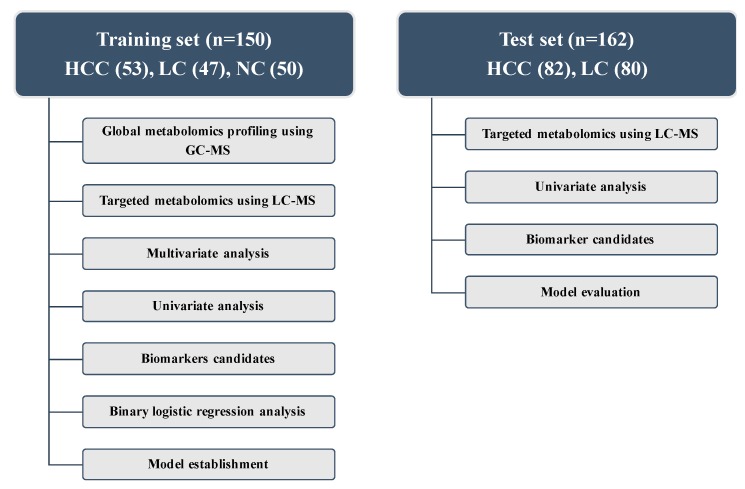
Design of the study and description of the training and test sets. HCC, hepatocellular carcinoma; LC, liver cirrhosis; NC, normal control; LC-MS, liquid chromatography-mass spectrometry; GC-MS, gas chromatography-mass spectrometry.

The workflow of metabolic profiling is shown in Figure 1. To obtain a broad range of metabolic candidates, we used two different ion-separating techniques, namely GC-MS and LC-MS, in the training set. The LC-MS technique was used in the test set to validate the biomarkers. 

As shown in Figure S1a, metabolic features obtained from GC-MS analysis were subjected to unsupervised pattern recognition analysis. In the training set, metabolic biomarkers differentiating HCC from cirrhosis were also compared with those of healthy subjects, thereby allowing the selection of further distinctive marker candidates. The first two principal components of a principal component analysis (PCA) score plot demonstrated 25.7% accuracy in the training set; Figure S1a illustrates the clustered groups. Through statistical analysis of the training set, 27 metabolites were identified, as shown in Table S1. As this study aimed to provide a biomarker panel that could be used with a statistical formula, these metabolites were quantified; therefore, metabolic candidates from the multivariate analysis were subsequently quantified. Among them, 18 metabolites were finally identified as significantly different among the three groups. An exception was citric acid, which exhibited a wide range of standard error, as shown in Table S2.

For targeted analysis, 188 metabolites were quantified based on LC-MS analyses. This technique provides quantified data of 40 acylcarnitines; 42 amino acids, biogenic amines, and monosaccharides; 15 sphingolipids; and 90 glycerophospholipids for each sample. Targeted analyses were repeated for both sets, as shown in Figure 1.

As GC-MS and LC-MS-based analyses are both specified to gather a large number of compounds simultaneously, multivariate and univariate analyses were performed with the false discovery rate (FDR) adjustment. Metabolites with variable important in projection (VIP) scores were analyzed using supervised pattern recognition analysis (PLS-DA), as shown in Figure S1b. The PLS-DA model demonstrated 18.6% accuracy when comparing HCC with cirrhosis in the training set. FDR-adjusted *p*-values of less than 0.05 and VIP scores greater than 1.0 were used to include variables for logistic regression. 

### 2.3. Potential Metabolic Biomarkers for HCC

**Figure 2:**
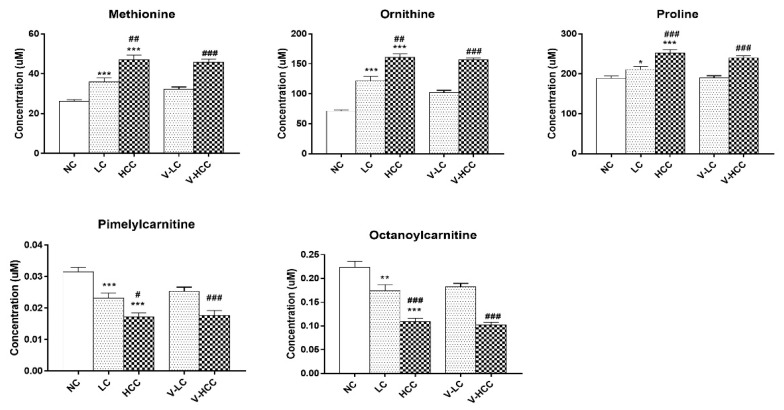
Comparisons among metabolic biomarkers. Bar graphs represent the concentration of individual metabolic biomarkers, namely methionine, ornithine, proline, pimelylcarnitine, and octanoylcarnitine. All the metabolites have VIP scores >1.0. FDR-adjusted *p*-values * < 0.05, ** < 0.001, or *** < 0.0001 represent a statistical significance compared with NC group. FDR-adjusted *p*-values # < 0.05, ## < 0.001, or ### < 0.0001 represent a statistical significance with LC or V-LC group. NC, LC, and HCC belong to the training set. V-LC and V-HCC belong to the test set. V-LC, liver cirrhosis in test set; V-HCC, hepatocellular carcinoma in test set. HCC, hepatocellular carcinoma; LC, liver cirrhosis; NC, normal control; VIP, variable important in projection; FDR, false discovery rate.

To identify potentially useful biomarkers, further assessment of biomarkers that simultaneously maintained the same change in trends compared to those of the test set was performed. To distinguish HCC from cirrhosis, binary logistic regression analysis and an optimized algorithm of the backward stepwise method were employed to construct the best model using these retained metabolic biomarkers. Ultimately, five metabolites were retained, namely methionine, ornithine, proline, pimelylcarnitine, and octanoylcarnitine, as shown in Figure 2. These five metabolites demonstrated statistical significance of FDR-adjusted *p*-values during analysis of variance (ANOVA) testing in the training set. They were also distinct from those of healthy controls.

The serum levels of methionine, ornithine, and proline progressively increased with disease severity, whereas those of pimelylcarnitine and octanoylcarnitine decreased. Notably, the concentrations of these metabolites were similar in the test and training sets. The metabolite concentrations in all the sub-groups are listed in Table S3. Therefore, the combination of these five metabolic biomarkers was ideal for distinguishing patients with HCC from those with cirrhosis. The model for the early detection of HCC was constructed as follows:

logit [P] = EXP (−1.716 + 0.035[Methionine] + 0.002[Proline] + 0.009[Ornithine] + 24.584[Pimelylcarnitine] − 14.088[Octanoylcarnitine])/1 + EXP (−1.716 + 0.035[Methionine] + 0.002[Proline] + 0.009[Ornithine] + 24.584[Pimelylcarnitine] − 14.088[Octanoylcarnitine])

**Figure 3:**
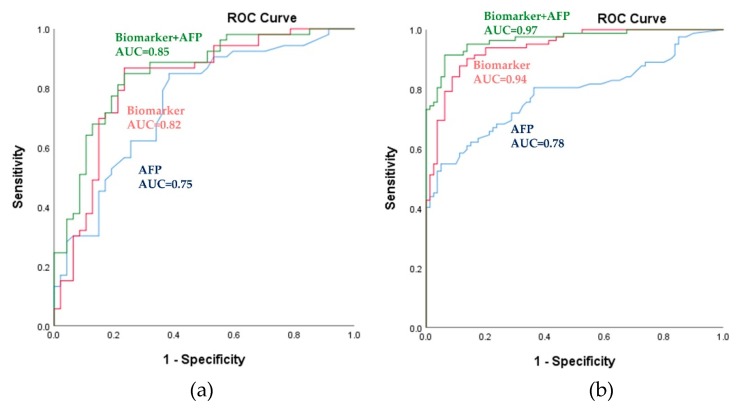
Power of the biomarker panel to discriminate HCC from LC, as assessed using the logistic regression model. (**a**) Receiver operating characteristic (ROC) curves for the training set. (**b**) ROC curves for the validation set. The blue-, red-, and green-colored lines indicate AFP, metabolic biomarkers, and metabolic biomarkers with AFP, respectively. AUC, area under the receiver operating curve; HCC, hepatocellular carcinoma; LC, liver cirrhosis; AFP, α-fetoprotein.

In this equation, [P = HCC] is the predicted probability of HCC by this panel, [metabolite] refers to the serum concentrations of metabolites. The panel of metabolic biomarkers showed significantly better performance compared to that of AFP, as indicated by an area under the receiver operating curve (AUC) of 0.82 (95% CI (confidence interval): 0.73–0.91) vs. 0.75 (95% CI: 0.65–0.85) in the training set; the results are shown in Figure 3a and Table 2. Combining the metabolic biomarkers with AFP increased the AUC value to 0.85. The performance of the metabolic biomarkers was further validated in the test set; this confirmed their reliability and higher predictability. As shown in Figure 3b and Table 2, the biomarkers also significantly outperformed AFP in the test set (AUC: 0.94; 95% CI: 0.91–0.98 vs. AUC: 0.78; 95% CI: 0.71–0.85). Combining AFP with the biomarkers slightly increased the AUC value in the test set to 0.97. In the training set, at the cutoff value of 0.49, the sensitivity and specificity were 79.20% and 78.70%, respectively, as shown in Table 2. In the test set, the sensitivity and specificity increased to 82.70% and 91.30%, respectively. As shown in Table 1 and Figure S2, AFP distinguished between HCC and healthy subjects with higher accuracy than it did between HCC and cirrhosis, with an AUC of 0.98 (95% CI: 0.96–1.00). Also, biomarkers clearly predicted HCC, with an AUC of 0.99 (95% CI: 0.98–1.00) and combining the biomarkers with AFP revealed 1.00 (95% CI: 1.00–1.00). Pearson correlation coefficients between differential metabolite contents and AFP demonstrated no significant correlation except for pimelylcarnitine, which showed a correlation coefficient of −0.2 and *p*-value of 0.042 in the training set only (data not shown). These findings suggest that the metabolic panel has a strong potential to be useful, displaying superior predictability compared to that of AFP for the discrimination of HCC.

### 2.4. Pathway Enrichment Analysis

**Figure 4:**
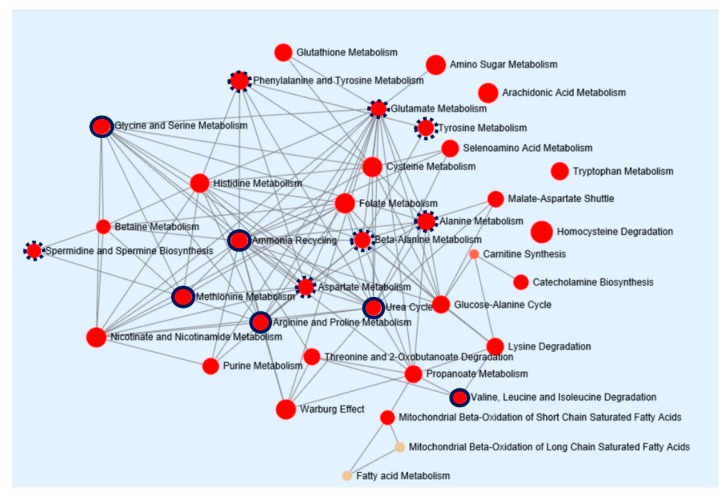
Pathway enrichment analysis of metabolic biomarkers in HCC. The network map represents pathways strongly correlated to metabolic dysfunction in HCC. Enrichment pathway analysis was based on the Kyoto Encyclopedia of Genes and Genomes database. All the represented metabolic pathways have FDR-adjusted *p*-values of less than 0.001. The sizes of the red circle indicate fold enrichment. Solid and dotted red circles represent metabolic pathways with more than four and three metabolic hits, respectively. HCC, hepatocellular carcinoma; FDR, false discovery rate.

To provide biological relevance between HCC and cirrhosis, pathway enrichment analysis was performed. Concentrations of 18 metabolites, identified from the global metabolomic profiling, were entered as input data. Metabolic pathway topological analysis measures the centrality in the differential correlation between cirrhosis and HCC. As shown in Figure 4, HCC-induced metabolic perturbations were strongly related to ammonia recycling, the urea cycle, and the metabolism of glycine and serine, methionine, and arginine and proline. In addition, the metabolism of glutamate, tyrosine, alanine, beta-alanine, and aspartate and the biosynthesis of spermidine and spermine were also associated with changes in metabolites between HCC and cirrhosis. The power of metabolic pathways, shown in Figure 4, was approved with FDR-adjusted *p*-values of less than 0.001 and Holm *p*-values of less than 0.05. An overview of this pathway enrichment, including fold enrichment, and its power are presented in Figure S3. These results indicate that compared with healthy subjects and patients with cirrhosis, patients with HCC may have more defects in ammonia recycling, the urea cycle, and amino acid metabolism.

## 3. Discussion

This study was designed to discriminate very early or early HCC from cirrhosis. We found a serum biomarker panel that included five metabolites, namely methionine, proline, ornithine, pimelylcarnitine, and octanoylcarnitine. Compared with serum AFP levels, this panel provided a higher accuracy for diagnosing HCC. In addition, the findings suggested that compared with that of cirrhosis, the development of HCC was more strongly associated with the dysregulation of ammonia recycling, the urea cycle, and amino acid metabolism.

AFP is the widely used traditional biomarker for HCC. However, AFP may also be expressed in certain pathological conditions, including chronic liver diseases, germ cell tumors, and gastric cancer [12,13,14]. Several studies that evaluated the diagnostic utility of AFP suggested that elevated serum levels of AFP (>20 ng/mL) correlated with an increased risk of HCC [15]. Despite the excellent sensitivity of AFP, its specificity remains low [11,16,17]. The low specificity of AFP as a biomarker for surveillance for HCC may be explained by several factors, such as the transient rise in AFP levels with exacerbations of hepatitis among patients with cirrhosis or chronic liver disease, flares of underlying liver disease such as hepatitis B or C virus infections, and the development of cancer. The use of AFP in clinical practice is therefore limited. The AFP cutoff value in our cohort was 0.46 (3.4 ng/mL), which was lower than that in literature reports. One patient with HCC had an AFP level of 65,632 ng/mL. However, the mean AFP levels for HCC in the training and test sets were 6.2 ng/mL and 10.3 ng/mL, respectively. AFP demonstrated relatively good sensitivity in the training set at 84.90%. However, the specificity was 61.70%. It also did not provide accurate results in the test set, with sensitivity and specificity of 74.10% and 67.50%, respectively. As mentioned earlier, the serum AFP level may not be reliable for the detection of HCC in the early stages; moreover, it appears to be more inaccurate among patients with cirrhosis. This may explain why it is unsuitable as a biomarker when used alone. Thus, a new set of biomarkers is much needed in this regard. 

GC- and LC-MS based metabolomics are powerful tools that have been used to discover novel circulating biomarkers for many diseases [5,6,7]. In the present study, to preserve active metabolites with distinct features as far as practicable, we combined both differentiated ion-separating techniques. The serum levels of any compounds demonstrating statistical significance were quantified to provide a more valid and reproducible prediction model for HCC. Notably, compounds identified on the multivariate and univariate analyses using GC-MS-based global metabolomic profiling overlapped with those of LC-MS-based targeted metabolomics. These metabolites included methionine, proline, and ornithine. Therefore, these markers may provide better differentiation of HCC.

Dysregulation of amino acid metabolism in liver disease has been long discussed. In particular, this study demonstrated changes in branched chain amino acid (BCAA) metabolism. BCAAs affect various bioprocesses, including energy metabolism and proliferation of hepatocytes [18,19]. Many studies have reported on the role of BCAAs in signaling pathways. Furthermore, BCAAs regulate glucose and lipid metabolism through the Phosphatidylinositol 3-kinases (PI3K)-Akt pathway and may also drive the mTOR (the mammalian target of rapamycin) pathway to regulate protein synthesis [20,21,22,23,24]. Reports also suggest that supplementation with those essential amino acids may improve general health and even prevent cancer [20,25]. However, there is also increasing evidence to suggest that higher BCAA levels promote cancer growth, since tumors use BCAAs as an energy source [25]. The current findings suggest that amino acids that were statistically significant amino acids in the analysis, namely methionine, ornithine, proline, threonine, methionine sulfoxide, and tyrosine, gradually increased during the transition from the healthy state to HCC. None of the patients in either training set or test set had hepatic encephalopathy and were treated with BCAA-enriched supplements.

In addition, the enrichment pathway analysis also suggested an association between ammonia recycling and the urea cycle. Waste ammonia, a byproduct of amino acid catabolism, must be excreted via the urea cycle [26]. These conversions mainly occur in the liver, where urea is produced and released. Amino acids such as glutamic acid, ornithine, asparagine, histidine, and aspartic acid are important intermediates in the urea cycle [27,28]; compared with patients with cirrhosis, patients with HCC had significantly enhanced amino acid levels. In normal physiological states, the urea cycle plays a major role in hepatic nitrogen disposal. There is mounting evidence that enzymes involved in the urea cycle, including two mitochondrial (carbamoyl phosphate synthase 1 and ornithine transcarbamylase) and three cytosolic (argininosuccinate synthase, argininosuccinate lyase, and arginase) enzymes, are differentially expressed in the liver. This enables the synthesis of urea cycle intermediates from nitrogen for cellular needs [29,30]. This may also explain anabolism in cancer cells, which is mediated by the rewiring of the urea cycle. This enables cancer cells to modulate metabolic resources, maximize biosynthesis, and economize carbon sources and nitrogen wastes. Moreover, because methionine metabolism mainly occurs in hepatocytes, methionine metabolism is also closely related to liver disease [31]. In patients with cirrhosis and HCC, the level of methionine adenosyltransferase (MAT1A), a crucial gene involved in the methionine metabolism, is downregulated, thereby leading to hypermethioninemia and reduced hepatic glutathione concentrations. Therefore, aberrant expression of genes involved in the methionine metabolism could explain the excessive accumulation of circulating methionine in patients with liver disease [31].

The alteration of serum acylcarnitine levels is another noticeable observation in this study. Through targeted metabolomic analysis, we quantified acylcarnitines from short- to long-chain fatty acids; interestingly, medium-chain acylcarnitines, such as pimelylcarnitine and octanoylcarnitine, were gradually diminished in patients with HCC. Acylcarnitines are also closely related to physiological activities and are a key source of energy metabolism in hepatocytes. Fatty acids conjugated with acylcarnitines are subjected to beta-oxidation in the hepatic mitochondria [28,32]. To traverse the mitochondrial matrix, long-chain acylcarnitines require specific transferases, such as carnitine/acylcarnitine translocase and carnitine palmitoyl transferase. However, acylcarnitines with medium-chain fatty acids can freely pass through the mitochondrial membranes and are utilized to produce fuel for energy [29,33]. Previous studies demonstrated that carnitine palmitoyltransferase 2 (CPT 2) expression is downregulated, thereby reducing hepatic beta-oxidation of fatty acids in patients with HCC [34,35]. A lack of CPT 2 expression ultimately leads to an increased level of long-chain acylcarnitines and reduced level of short- and medium-chain acylcarnitines in circulation [34,35]. Therefore, we speculate that due to the rapid transport of medium-chain acylcarnitines into the liver, the cancer cells may utilize this energy source to proliferate. This may explain the lower levels in patients with HCC than in those with cirrhosis.

## 4. Materials and Methods 

### 4.1. Study Population

This study included patients who were enrolled in two prospective cohorts at the Seoul National University Hospital (Seoul, Republic of Korea). The training set comprised 53 patients with very early or early HCC based on the Barcelona Clinic Liver Cancer staging system [1], 47 patients with cirrhosis, and 50 healthy controls enrolled between January 2014 and August 2017 as part of an ongoing study to identify biomarkers associated with the prognosis of HCC. The test set for the validation of the biomarker signatures consisted of 82 patients with very early or early HCC and 80 patients with cirrhosis from an independent study evaluating the metagenomics profiling of HCC between April 2017 and October 2018. 

HCC was mostly diagnosed based on the noninvasive criteria of an international guideline [1,2]. Cirrhosis was diagnosed based on either histological or clinical findings [36]. The control group included patients with no clinical or imaging evidence of liver disease. Clinical and biochemical data were derived from either the registry databases or medical records. Overnight fasting serum samples were obtained from all patients and were stored at −80 °C. 

This study was approved by the institutional review board of Seoul National University Hospital (SNUH IRB No. 1704-021-843) and was conducted in accordance with the principles of the Declaration of Helsinki. All participants provided written informed consent. The Reporting Recommendations for Tumor Marker Prognostic Studies criteria were followed throughout the study [37].

### 4.2. Global Metabolomics Using Gas Chromatography Time-of-Flight Mass Spectrometry (GC-TOFMS)

The frozen serum samples were thawed at 4 °C, and 50 µL of each sample was diluted with 1 mL of an acetonitrile/isopropanol/water (3:3:2) mixture; 400 µL of the supernatant was subsequently evaporated to complete dryness at room temperature and was then reconstituted with 50% acetonitrile to a volume of 400 µL. Centrifugation was then repeated for 5 minutes at 18,341× *g* at 4 °C, followed by evaporation of the supernatant. The residue was reconstituted with methoxyamine dissolved in pyridine and a mixture of N-methyl-N-(trimethylsilyl) trifluoroacetamide and fatty acid methyl esters. Aliquots (1 µL) were then injected in splitless mode into an Agilent 7890 A gas chromatograph coupled with a Pegasus HT time-of-flight mass spectrometer (Leco Corporation, St. Joseph, MI, USA). The detailed experimental procedure has been described in our previous study [38]. Among 1292 metabolites detected using the global metabolomic profiling, compared with authentic compounds, unknown compounds and metabolites with different retention times were excluded, and the remaining values were statistically verified. After marker selection using statistical analyses, the concentration of the metabolites was quantified, as shown in Table S2.

### 4.3. Targeted Metabolomics Using Liquid Chromatography with Tandem Mass Spectrometry (LC-MS/MS)

A total of 180 metabolites were quantified using the AbsoluteIDQ® p180 kit (BIOCRATES Life Science AG, Innsbruck, Austria) with an LC-MS/MS system, allowing for concurrent high-throughput detection and quantification of metabolites in the serum samples. This platform was combined with direct flow injection (acylcarnitines and glycerophospholipids) and liquid chromatographic (amino acids and biogenic amines) mass spectrometric approaches. The protocol followed was in accordance with the manufacturer’s instructions. In brief, 10 μL of serum was transferred onto the 96-well kit plate containing the standard and isotope-labeled internal standards. After evaporating the serum sample under nitrogen gas, it was derivatized with phenylisothiocyanate reagent. The metabolites were then extracted using 5 mM ammonium acetate in methanol for standard and direct injection analysis mass spectrometry. The details of the LC-MS/MS analysis and data processing have been described in our previous report [39].

### 4.4. Statistical Analysis

Multivariate, univariate, and enrichment pathway analyses were conducted using the Metaboanalyst 4.0 tool. Normalized data based on the total sum of all detected ions were entered, and PCA was performed to examine differences in overall metabolite profiles among the groups. The multiple test problem was corrected by adjusting FDR using the Benjamini and Hochberg method. Concentrations of the selected metabolites were subjected to enrichment pathway analyses using Metaboanalyst 4.0 to meaningfully identify and interpret patterns of metabolic changes. Metabolites that were significantly different between LC and HCC were matched with predefined metabolite pathways from Kyoto Encyclopedia of Genes and Genomes (KEGG).

Quantified results, including newly identified metabolites from GC-TOFMS and compounds in the AbsoluteIDQ® p180 kit (BIOCRATES Life Sciences AG, Innsbruck, Austria), were subjected to partial least squares-discriminant analysis (PLS-DA) to maximize the distance between groups and to identify variables with important contributions to the classification of the variable important in projection (VIP). The permutation test was repeated 1000 times to assess the risk of overfitting for the model.

Metabolites with FDR-adjusted *p*-values less than 0.05 and VIP scores greater than 1.0 were reserved to construct the prediction model. The detailed procedure for marker selection is shown in Figure S4. For the comparison of categorical data, χ^2^ or Fisher’s exact test was used. Pearson correlation analysis and binary logistic regression were performed using the SPSS 25.0 software (IBM Corp., New York, USA) package.

## 5. Conclusions

In conclusion, global and targeted metabolomic profiling using GC-TOFMS- and LC-MS/MS-based analyses provided relatively wider metabolite coverage in this cohort. It also provided a metabolic biomarker panel of three amino acids (methionine, proline, ornithine) and two acylcarnitines (pimelylcarnitine and octanoylcarnitine). The findings validated this panel as a potential diagnostic tool for the early detection of HCC. Further large prospective studies with external validation are warranted to determine whether this metabolite panel may improve surveillance and management strategies in patients with HCC.

## Figures and Tables

**Table 1:** Clinical characteristics of the subjects in each set.

	Training Set				Test Set		
Variables	HCC	Cirrhosis	Healthy Controls	*p*-Value	HCC	Cirrhosis	*p*-Value
	(*n* = 53)	(*n* = 47)	(*n* = 50)		(*n* = 82)	(*n* = 80)	
**Age, years**	59.0 (54.5–65.5)	60.0 (55.0–65.0)	51.0 (38.8–58.3)	0.73	62.0 (55.0–68.3)	59.0 (52.0–66.0)	0.09
**Male**	35 (66.0%)	34 (72.3%)	25 (50.0%)	1.00	57 (69.5%)	54 (67.5%)	1.00
**Etiology of liver disease**							
				0.002			0.03
HBV	53 (100.0%)	39 (83.0%)	-		51 (62.1%)	55 (68.8%)	
HCV	0	6 (12.8%)	-		13 (15.9%)	3 (3.8%)	
Non-viral	0	2 (4.2%)	-		18 (22.0%)	22 (27.5%)	
**Child-Pugh class**				0.10			1.00
A	52 (98.1%)	42 (89.4%)	-		78 (95.1%)	76 (95.0%)	
B	1 (1.9%)	5 (10.6%)	-		4 (3.7%)	4 (5.0%)	
**α-fetoprotein, ng/mL, median (IQR)**	6.2 (3.9–21.3)	2.8 (2.2–5.6)	1.4 (1.0–2.0)	<0.001	10.3 (4.2–33.6)	3.3 (2.1–4.9)	<0.001
**BCLC stage**							
Very early	28 (52.8%)				46 (56.1%)		
Early	25 (47.2%)				36 (43.9%)		

Data are presented as medians with interquartile ranges (IQRs) or numbers (%), unless otherwise indicated. AJCC, the American Joint Committee on Cancer; HCC, hepatocellular carcinoma; BCLC, Barcelona Clinic Liver Cancer staging system; HBV, hepatitis B virus; HCV, hepatitis C virus.

**Table 2:** Test performance characteristics for the biomarker signature from the training and test set.

Set	Group	Data Set	AUC (95% CI)	Biomarker Cutoff	Sensitivity %	Specificity %
Training	HCC vs NC	AFP	0.98 (0.96–1.00)	0.26	94.30	92.00
		Biomarker	0.99 (0.98–1.00)	0.48	96.20	98.00
		Biomarker+AFP	1.00 (1.00–1.00)	0.50	100.00	100.00
Training	HCC vs LC	AFP	0.75 (0.65–0.85)	0.46	84.90	61.70
		Biomarker	0.82 (0.73–0.91)	0.49	79.20	78.70
		Biomarker+AFP	0.85 (0.76–0.93)	0.52	81.10	78.70
Test	HCC vs LC	AFP	0.78 (0.71–0.85)	0.46	74.10	67.50
		Biomarker	0.94 (0.91–0.98)	0.49	82.70	91.30
		Biomarker+AFP	0.97 (0.71–0.85)	0.52	82.70	95.00

AUC, area under the receiver operating curve; CI, confidence interval; HCC, hepatocellular carcinoma; LC, liver cirrhosis; NC, normal control; AFP, α-fetoprotein.

## Supplementary Materials

The following are available online at https://www.mdpi.com/2072-6694/11/10/1497/s1. Figure S1: Identification of potential metabolic biomarkers for the diagnosis of early HCC in the training set, Figure S2. The power of biomarker panels to discriminate hepatocellular patients from NC on the logistic regression model, Figure S3. Pathway enrichment overview of metabolic biomarkers in hepatocellular carcinoma, Figure S4. Procedure for marker selection from GC-TOFMS and LC-MSMS-based data, Table S1. List of metabolic candidates observed from GC-MS analysis in training set, Table S2. Concentration of metabolic biomarker candidates in training set, Table S3. Concentration of biomarker panel in training and test set
