# Supplementary material for: Comprehensive Metabolomic Search for Biomarkers to Differentiate Early Stage Hepatocellular Carcinoma from Cirrhosis
Source: Cancers (Basel). 2019 Oct 6;11(10):1497. doi: 10.3390/cancers11101497 (PMC6826937; doi:10.3390/cancers11101497)
Supplement: Supplementary file 1 [file cancers-11-01497-s001.pdf]

Supplementary Materials

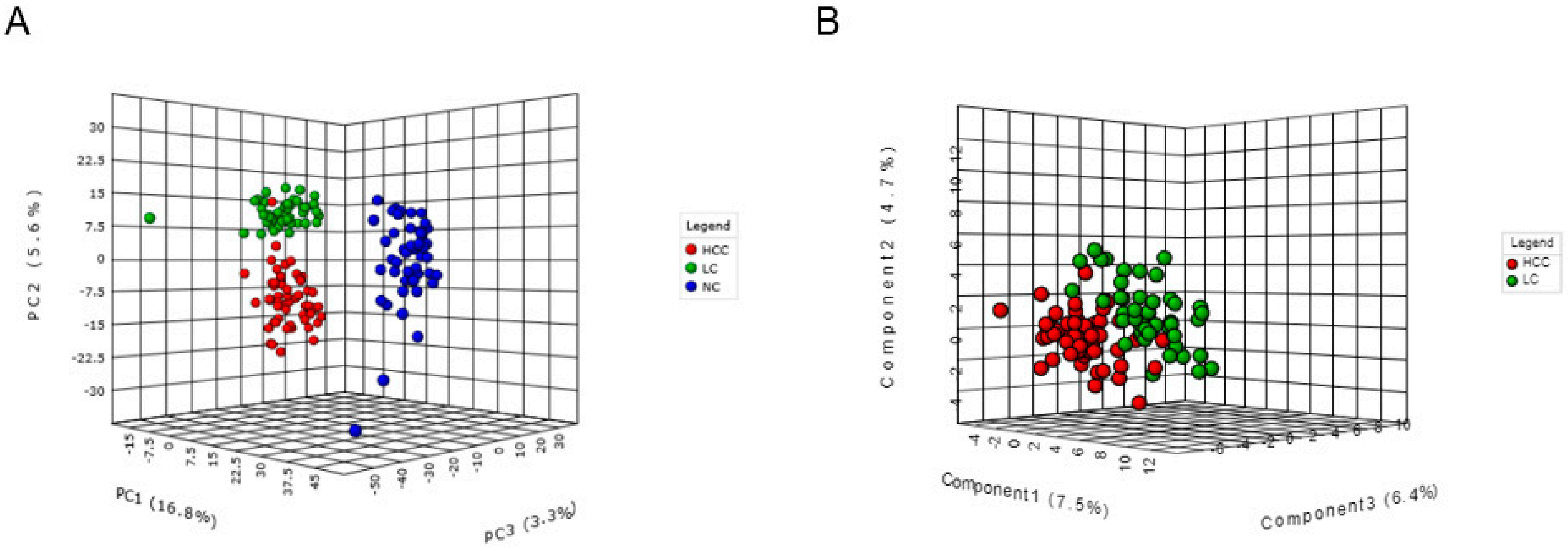

Figure S1. Identification of potential metabolic biomarkers for the diagnosis of early HCC in the training set. (**a**) score plot of unsupervised multivariate analysis (PCA) including HCC, LC, and NC. (**b**) score plot of supervised multivariate analysis (PLS-DA) to discriminate metabolites of HCC from LC. The red, green, and blue circles indicate patients with HCC, LC, and NC, respectively.

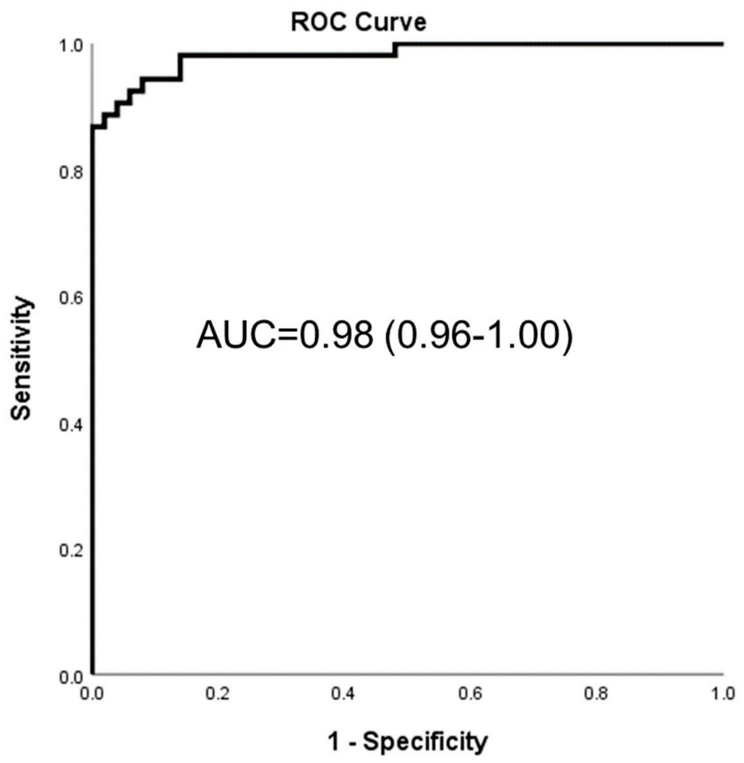

Figure S2. The power of biomarker panels to discriminate hepatocellular patients from NC on the logistic regression model. A Receiver operating characteristic curves for the training set.

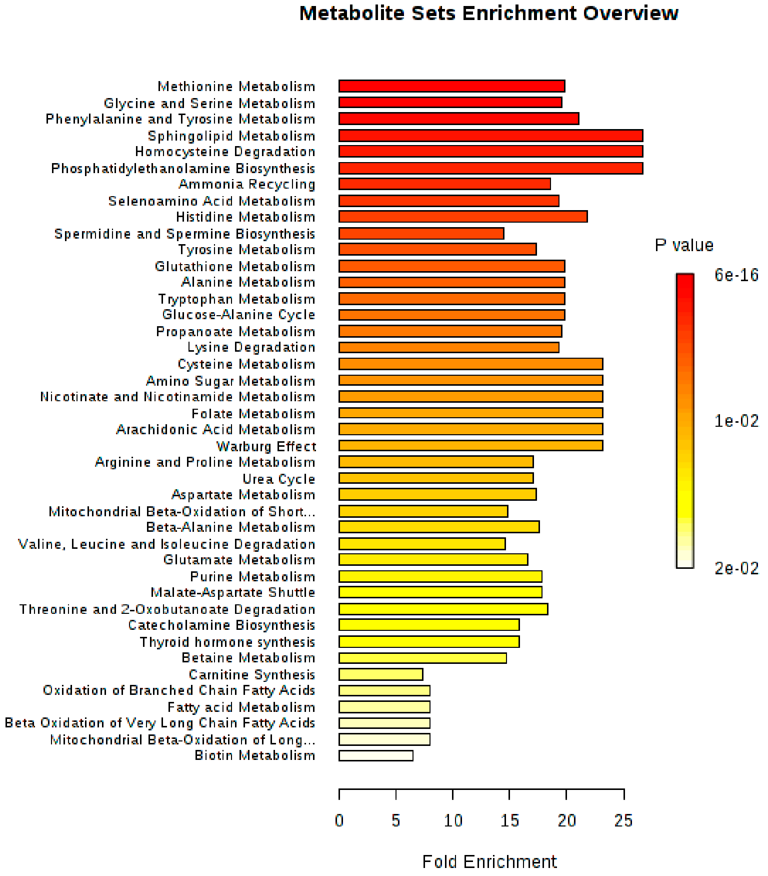

Figure S3. Pathway enrichment overview of metabolic biomarkers in hepatocellular carcinoma. The width of each bar graph indicates the size of fold enrichment. The colors on the bar graph represent the *p*-value of each metabolic pathway.

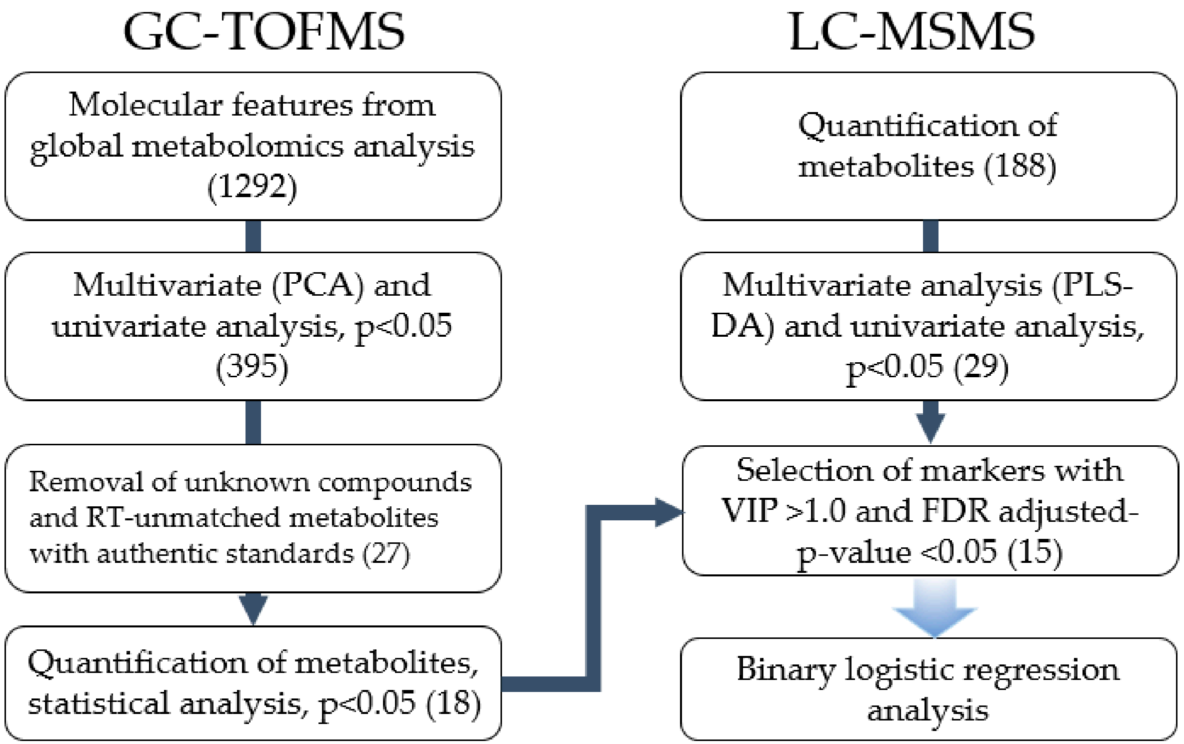

Figure S4. Procedure for marker selection from GC-TOFMS and LC-MSMS-based data.

Table S1. List of metabolic candidates observed from GC-MS analysis in training set.

Analytes	Chemical Formula	Unique Mass	RT	Similarity	Reverse	Probability	p.value	FDR	MSI level
L-Valine	C5H11NO2	72	284.1	890	890	9361	1.18E-17	4.61E-17	1
L-Alanine	C3H7NO2	116	291.45	906	906	8202	8.25E-08	1.82E-07	1
L-Proline	C5H9NO2	70	325.15	859	859	9723	1.01E-03	1.74E-03	1
L-Isoleucine	C6H13NO2	86	325.45	766	773	7573	1.95E-21	1.32E-20	1
Picolinic acid	C6H5NO2	180	389.65	784	821	7981	3.87E-11	9.97E-11	1
Serine	C3H7NO3	204	404.05	934	937	9050	1.41E-06	2.93E-06	1
L-Threonine	C4H9NO3	218	415.35	880	880	7651	1.60E-12	4.41E-12	1
Aminoisobutyric acid	C4H9NO2	174	444.55	870	872	7647	5.43E-13	1.59E-12	1
L-Aspartic acid	C4H7NO4	232	465.95	847	878	3589	8.04E-13	2.28E-12	1
L-Methionine	C5H11NO2S	176	466.7	893	898	9803	3.55E-09	8.25E-09	1
Propanoic acid	C3H6O2	71	494.65	886	886	3978	6.90E-08	1.53E-07	1
L-Glutamic acid	C5H9NO4	246	501.05	797	797	3114	9.11E-16	3.16E-15	1
Phenylalanine	9H11NO2	218	506.7	901	901	8148	2.20E-10	5.40E-10	1
L-Asparagine	C4H8N2O3	116	520.1	921	936	9139	1.33E-08	3.00E-08	1
L-Ornithine	C5H12N2O2	142	568.75	945	947	9828	1.18E-16	4.33E-16	1
Citric acid	C6H8O7	273	569.65	854	865	8828	3.97E-11	1.02E-10	1
L-Lysine	C6H14N2O2	174	599.95	919	919	9871	4.08E-05	7.75E-05	1
L-Histidine	C6H9N3O2	154	601.95	723	727	1856	1.59E-02	2.38E-02	1
L-Tyrosine	C9H11NO3	218	606.35	862	862	6196	4.10E-21	2.34E-20	1
L-Tryptophan	C11H12N2O2	202	687.8	935	935	8231	7.80E-13	2.23E-12	1
L-Cystine	C6H12N2O4S2	218	705.1	942	945	9871	2.58E-35	5.38E-34	1
Azelaic acid	C9H16O4	317	558.35	870	870	9099	5.79E-45	2.23E-43	1
Linoleic acid	C18H32O2	337	678.75	966	966	9891	6.35E-06	1.28E-05	1
Inosine	C10H12N4O5	230	772.7	718	718	5508	3.83E-40	1.20E-38	1
Arachidonic acid	C20H32O2	80	720.15	845	856	8980	2.31E-11	6.05E-11	1
Oleic acid	C18H34O2	156	681.9	867	867	9287	5.35E-04	9.52E-04	1
Uric acid	C5H4N4O3	339	679.6	983	983	9875	1.48E-05	2.87E-05	1

RT, retention time; FDR, false discovery rate; MSI, metabolomics standards initiative

Table S2. Concentration of metabolic biomarker candidates in training set.

Metabolite	NC	LC	HCC	Anova	Bonferroni
Phenylalanine	111.98 ± 2.08	91.92 ± 3.15	115.44 ± 3.54	***	###,†††
Proline	188.76 ± 5.74	210.89 ± 7.60	252.15 ± 8.66	***	$$$,†††
Valine	238.60 ± 5.21	239.66 ± 6.28	271.64 ± 7.81	***	$$,††
Isoleucine	88.33 ± 2.62	95.05 ± 3.60	117.00 ± 5.26	***	$$$,††
Alanine	406.50 ± 10.21	375.72 ± 12.63	444.15 ± 13.76	***	†††
Aspartic acid	61.85 ± 2.42	22.82 ± 1.98	31.54 ± 2.13	***	###,$$$,†
Asparagine	73.68 ± 2.11	65.81 ± 2.63	85.69 ± 3.56	***	$,†††
Threonine	132.47 ± 4.21	138.50 ± 3.71	168.88 ± 4.81	***	$$$,†††
Tryptophan	67.81 ± 1.62	81.60 ± 3.46	77.88 ± 1.80	**	##
Tyrosine	67.97 ± 1.36	110.52 ± 3.82	110.01 ± 3.71	***	###,$$$
Ornithine	71.23 ± 2.20	121.79 ± 7.39	160.64 ± 6.02	***	###,$$$,†††
Histidine	113.16 ± 2.04	101.30 ± 2.17	114.23 ± 2.23	***	##,$$$
Methionine	26.15 ± 0.89	35.85 ± 2.05	47.05 ± 2.35	***	##,$$$,†††
Serine	205.38 ± 5.44	159.60 ± 5.51	213.94 ± 7.30	***	###,†††
Glutamic acid	193.30 ± 6.88	85.55 ± 10.27	148.36 ± 9.32	***	###,$$,†††
Azelaic_acid	78.45 ± 3.96	1.68 ± 0.29	2.04 ± 0.34	***	###,$$$
Citric_acid	0.02 ± 0.00	0.05 ± 0.01	0.776 ± 0.36	*	n.s.
Linoleic_acid	692.47 ± 70.71	189.46 ± 44.44	373.77 ± 156.54	**	##

Values are expressed as mean concentration (uM) ± SE or frequency one way anova * p < 0.05; ** p < 0.01; *** p < 0.001 when compared to NC vs LC # p < 0.05; ## p < 0.01; ### p < 0.001 when compared to NC vs HCC $ p < 0.05; $$ p < 0.01; $$$ p < 0.001 when compared to non-NC groups (LC and HCC) †p < 0.05; †† p < 0.01; ††† p < 0.001 Values are expressed as mean ± SE or frequency

Table S3. Concentration of biomarker panel in training and test set.

Metabolite	NC			LC			HCC			Test-LC			Test-HCC		
	**Mean**	**SD**	**N**	**Mean**	**SD**	**N**	**Mean**	**SD**	**N**	**Mean**	**SD**	**N**	**Mean**	**SD**	**N**
Methionine	26.15	6.29	50	35.85	14.04	47	47.05	17.08	53	32.30	9.40	80	45.82	14.67	82
Ornithine	71.23	15.58	50	121.79	50.63	47	160.64	43.82	53	102.30	29.30	80	156.70	27.97	82
Proline	188.76	40.60	50	210.89	52.09	47	252.15	63.08	53	190.50	41.86	80	239.60	51.54	82
Pimelylcarnitine (C7-DC)	0.03	0.01	50	0.02	0.01	47	0.02	0.01	53	0.03	0.01	80	0.02	0.01	82
Octanoylcarnitine (C8)	0.22	0.09	50	0.17	0.09	47	0.11	0.05	53	0.18	0.07	80	0.10	0.05	82

Data are presented with mean ± standard deviation. NC, normal control; LC, liver cirrhosis; HCC, hepatocellular carcinoma.
